# A Few Remarks on the Role of the American Dentists in Paris during the Great War*Read before the American Dental Society of Europe, London, England, July 22, 1922.

**Published:** 1923-03

**Authors:** Field Robinson


### A FEW REMARKS ON THE ROLE OF THE AMERICAN DENTISTS IN PARIS DURING THE GREAT WAR *

BY DR. FIELD ROBINSON

*Mr. President, Gentlemen:*

The Executive Committee has conferred upon me a great honor by requesting me to make a condensed report, or sort of *résumé,* of the work done by the American dentists in Paris during the period of hostilities—1914-1918. I appreciate that sincerely! And I fear that it will be impossible for me to do ample justice to the subject, or to those who set such admirable examples of voluntary devotion and service, spontaneously and energetically, as soon as the call on their sense of duty reached them. It is necessary for me to generalize as much as possible. Nevertheless, I can not abstain from rendering a tribute of esteem and praise, which is more than due, to some in particular, and consequently will be compelled to mention names, for which I apologize, as I know that the natural modesty of these men will be offended somewhat by my doing so.

Many amongst you will remember the intense feeling of anxiety and apprehension concerning probable international events which pervaded the atmosphere of our meeting in Paris in 1914, and the rapid flight towards home of those who resided in possible belligerent countries. The declaration of war between Germany and France justified these feelings. Those who remained in Paris began at once to consider how they could become useful, professionally, to those who were to become combatants. Their inquiries brought out the salient fact that the services of the dentist were not esteemed to be of much value. They found very slight, if any, apprecia-

* Read before the American Dental Society of Europe, London, England, July 22, 1922.

tion of the rôle the dentist could possibly fulfill. This state of things led to a period of disappointment, but the spirit of determination to be useful was there and, refusing to be daunted, they decided to seize any opportunities which might present, to begin, in no matter how humble a way, the work which they were convinced would impose its own value.

### ORGANIZATION OF THE AMERICAN AMBULANCE

The first chance came when the American Ambassador in Paris, Mr. Myron T. Herrick, with the assistance of the Medical Board of the American Hospital and willing American civilians, formed a committee to consider the organization of a special hospital, with an ambulance service which it was proposed to offer as an American contribution to France in her hour of trouble. This was to be an American offering under the auspices of the American Hospital and under the direction of the Medical Board of that institution. Mrs. Herrick organized at the same time the Woman's Committee of the American Ambulance of Paris which was offered to the French Military Government for the aid of wounded soldiers. The huge building, the Lycée Pasteur in Neuilly, was obtained from the French Government, where over a thousand beds could be placed.

### DRS. GEO. B. HAYES AND W. S. DAVENPORT

On August 7th (the sixth day of mobilization) Dr. George B. Hayes's voluntary offer, made on the 4th of August, to organize the dental service was accepted and he was duly elected a member of the Medical Board. This is the first official recognition of the possible use of the dentist recorded, and from this there grew up one of the finest organizations of our special work which the war produced in and about Paris. This distinction of being the first to have a dental surgeon on the Medical Board of a military hospital belongs to this American Ambulance. Dr. William S. Davenport was appointed to act conjointly with Dr. Hayes to complete the installation of the dental department, installation composed of their own chairs, engines, instruments and supplies. After the first fortnight, the occupants of three hundred beds came under their partial control. Prophylactic work, with cleaning up infected mouths, treatment of cases requiring immediate dental attention for those unable yet to go to the dental surgery, formed the commencement of their self-imposed duties.

We Britishers owe a huge debt of gratitude to these two colleagues and the American Ambulance for the efficient and useful work which they did for our men, who had no dental surgeons to attend to them and *ipso facto* no dental organization at that time.

The Dental Department of the American Ambulance at Neuilly soon became a center to which flocked not only cases for conservative dentistry, making of numerous dentures for those unable to masticate trench food through lack of natural teeth, etc., but also of wounds to the jaws and face, as colleagues and confreres were drawn to it to study its organization and to help them to establish for their own men similar refuges for those in distressful need of the dentist.

The fractured jaw cases increased in number daily and caused a severe test of physical endurance on Drs. Hayes and William Davenport. Ready help was at hand, however, and thanks to the much-appreciated and voluntary collaboration of Dr. Ch. Hotz, Professor Potter of Harvard University, Dr. Darcissac, Dr. Ortion, Dr. Stuhl, Dr. Georges Roussel, who contributed during longer or shorter periods their active assistance, the high standard of proficiency in the work done and the services rendered gave to the American Ambulance a position which was due to it and which served as an example we all tried to follow.

Drs. Le Cron, senior and junior, Dr. Roberts from amongst you, also gave three months of their time and skill.

I think therefore there is every reason to be proud of the individual efforts of the promoters of the American Ambulance and of the Dental Department in particular. The French Government bestowed the honor of Chevalier de la Légion d'Honneur on Dr. Hayes and Dr. W. S. Davenport and the American Government the D. S. Medal on Dr. Hayes in recognition and appreciation of his valuable services.

DR. VALADIER

Towards the end of September, 1914, I was appointed Senior Dental Surgeon of the British Red Cross Society of Paris and surrounding districts. During one of my visits to the Dental Depôts in Paris, with the object in view of organizing a dental center for our men, fortuitously I met Dr. Valadier. In reply to my questions concerning what he purposed doing, he informed me that he would be only too glad to do anything he possibly could do as a dentist. At that time calls were being made urgently from the British Headquarters, then at Villeneuve-St. Georges, for a dental surgeon. I asked Dr. Valadier whether he would offer his services to the British Red Cross and go wherever he would be sent by the Council to work for the British Army, emphasizing at the same time that he would have to provide his own equipment of instruments and material, etc. This he gladly agreed to do; consequently I requested him to call on the president of the committee at the headquarters of Paris branch of the British Red Cross Society (Dr. Leonard Robinson) that very afternoon, and to make a written engagement which would be submitted for approval at the meeting of the Council—as a voluntary dental surgeon. His offer was accepted. Straight away Dr. Valadier set to work to transform his own closed motor car into an ambulant dental surgery and provided himself with everything necessary for immediate active dental service. The following day he received orders to report to the Assistant Director of Medical Service, British Expeditionary Forces in France, stationed at Abbeville. There he found himself to be, strange as it may appear, the only dental surgeon attached to the army. From there he went to Poperinghe, Dieppe, Ypres and Boulogne. Active and energetic, determined to do his utmost for all who needed dental treatment, his efforts were particularly applied to allay the acute conditions which presented, and at the same time do what he could for fractured jaw cases. This help was gladly welcomed and the utility of the dentist imposed itself through his useful and efficient work. He commenced work in Boulogne in a room situated in the cellar of the Boulogne Casino—which was then No. 13 General Hospital, but the results obtained and the satisfaction given by his dental services to many of the superior officers permitted him to obtain, about the autumn of 1915, the sanction of the Medical Department for the construction of special dental huts. The amount of work done there in collaboration with a number of young dental surgeons, who joined him during 1915 and onwards (they then being Army Dental Surgeons), willing and ambitious to do all in their power, added to the rapidity with which soldiers who came for treatment were seen, cared for and discharged fit for duty. Also, the organization of the dental department attracted notice, and it was not long before the highest authorities recognized the value of the services rendered by the dentists, and promised their collaboration and assistance in extending the help of the dental surgeon. The Director of the Medical Service recommended and obtained for Dr. Valadier the honorary rank of Major in the Royal Army Medical Corps and Chief Dental Surgeon to the British armies in the field.

Independently of the establishment of the maxillo-facial department attached to this service, there was the extremely active prosthetic section where from 35 to 50 dentures were completed every second day, operative dentistry for preversation of teeth, etc., not attaining a proportionate level.

You all know that Dr. Valadier's services, given gratuitously, from October, 1914, to the end of hostilities, were rewarded by His British Majesty's Government by his being granted a knighthood and that he is now Sir Charles Valadier.

DRS. ISAAC B. AND RALPH DAVENPORT

Towards the end of December, 1914, I received, as Senior Dental Surgeon, British Red Cross Society, an appeal for help from Monsieur Georges Guyon, the administrator of the Hospital St. Vincent de Paul, Paris (Auxiliary Hospital No. 65), there being no dental surgeon attached to the hospital, and patients requiring immediate dental treatment. Knowing how ever ready to help Dr. Isaac B. Davenport and his son, Dr. Ralph Davenport were, I called upon them to solicit their professional services for this hospital. They very kindly accepted and at once began active service.

They organized a dental service for wounded and sick soldiers at this hospital, being in attendance from 8 a. m. to 12, from January, 1915, to January, 1918, without interruption and until the hospital closed after the end of the war. Their first operations were done at the bedside, but their good work had such beneficial effects, that a temporary office was established in the private bedroom of the director of the hospital, there being no other place free. This went on for a year or more, when, on account of the growing importance of the dental service, suitable and excellent quarters were attributed to the dental surgeons in a new dispensary, where not only the patients from this hospital received full treatment, but patients from about ten other auxiliary hospitals, at which no dentist attended, were welcomed in this dental service.

Over 900 patients received plain ordinary every day dentistry which enabled many of them to return to the fighting lines. The thoroughness of the work done was on a par with that of the private office. More than 150

plates were provided. The majority of the patients rarely had any sort of dentistry done for them, save extractions, but they soon appreciated the excellent results of conservative dentistry. All expenses, except use of premises, heat, light and one operating chair, were paid by Dr. Isaac Davenport.

When America came in, Dr. Ralph Davenport was attached to the American Army Dental Corps and was occupied at the Ambulance. His place was taken by Dr. William Davenport, who transferred his services from the American Ambulance, Dental Department, to this Auxiliary Hospital, thus rendering valuable help to his brother, Dr. Isaac Davenport.

Dr. Isaac Davenport was also associated with the Special American Hospital for wounds of the face and jaws, Hôspital Bénévole No. 16, founded largely by the raising of a large sum in America (nearly 500,000 francs) for the purpose and assurance of its maintenance. This was a fine and well-equipped hospital with 80 beds, excellent surgical operating rooms and a very large dental department with six operating chairs installed and six others ready to be installed; laboratories, radiography, etc., etc. Owing to the irascibility of character of the French chief surgeon and his marked disdain for the dentists, perhaps particularly for the American dentists, this promising institution was closed as the administrative committee entrusted with the funds considered that the objects and intentions of the donors were not, and could not be realized without a change of the chief surgeon, which was not granted by the military hospital authorities. The unused funds were either offered to the donors, or being refused by them, to the American Red Cross which, in its turn, donated the sum of 30,000 francs to the Ecole Dentaire de Paris for the Face and Jaw Service.

### DR. JOHN H. SPAULDING

Dr. John H. Spaulding was the President of the Committee of Organization of Hôpital Bénévole No. 16 bis and as such did all in his power to make it a success and of special advantage for those wounded in the face or jaws.

DR. F. J. WILSON

Dr. F. J. Wilson was appointed chief of the Dental Service in Ambulance B. of the American Hospital for French Wounded situated at Juilly (Seine-et-Marne), where valuable dental work was done to those who, passing through this kind of sorting hospital, were in dire need of temporary work, either by the aid of vulcanite splints or by wiring fractured portions of jaws in their normal position, retaining normal occlusion and giving the patients as much comfort as possible until something permanent could be done at a base hospital. Prophylactic work, fillings and extractions were done for all who needed same during their short stay in this hospital.

DR. ORTION

Dr. Wilson was aided by Dr. Ortion from October, 1915, to December, 1917, when Dr. Ortion volunteered with the American Expeditionary Forces and served until honorably discharged in June, 1919, after having been recommended three times.

DR. HALLY SMITH

A well-organized clinic was established by Dr. Hally Smith in the Citroën works converted into an ammunition factory producing shells, etc. Ordinary daily routine dentistry was freely given to the numerous civilians working there.

DR. ABRAHAM HIPWELL

Dr. Abraham Hipwell did all he could to be useful to the members of the Flying Corps and did voluntary work for the French children taken care of by the Suffragettes.

DR. FIELD ROBINSON

During the first days of October, 1914, I was nominated by the Council of the Paris Branch of the British Red

Cross as Senior Dental Surgeon and requested by the same to form a dental service for the use of the men of the British Expeditionary Forces in France who required dental treatment, there being at that period no Army Dental Surgeons attached to this Expeditionary Force. Our late and much regretted member, Dr. Ernest Burt, soon joined me as Assistant Dental Surgeon and thanks to the lively interest taken in this special work by the Council of the B. R. C. S. premises were provided at their General Headquarters where I established, entirely at my own cost, the complete outfit and materials forming the nucleus of a dental service which was destined to become of considerable importance. Dr. Valadier kindly lent me a chair and cuspidor which allowed us conjointly to tackle the ever-increasing number of patients needing immediate dental care and attention. The ultimate success of our enterprise was due, in a very great measure, to the sympathetic help and encouragement given to us by the Director General of the Medical Service of the British Expeditionary Forces in France, Sir Arthur Sloggett, he having recognized, with ready perspicacity, how useful our services might be. Dr. Hargreaves and Dr. Gick—both University of Pennsylvania graduates—rendered great help by their constant and assiduous application to their duties as Assistant Dental Surgeons, duties which only ended with the month of May, 1919.

My first experiences were similar to those encountered by Dr. George Hayes and Dr. Valadier. At No. 4 General Hospital, Versailles, I was plainly told that really the commanding officers of that hospital could not see what important use the dentist could be, and I was first offered the end of a corridor to establish, as best I could, my necessary installation, prior to the final appointment of a bathroom which, being well lighted, made an excellent office. The supplying of dentures to men who otherwise than their inability to masticate food, were physically fit and good fighting units, gave the first opportunity to prove that we were of some utility, inasmuch as these men invalidated temporarily through dental deficiencies were able to return to the front and take their places in the fighting line, and at a period when compulsory military service had not been voted and trained soldiers were scarce. Others, sent down from the front, who were constantly tortured by facial neuralgia of dental origin gave us another chance to destroy the apparently biased opinions held by the Army Medical Corps staff about the real need of the dentist and his special work, for these men showed their gratitude by their voiced appreciation of the treatment given and relief obtained thereby.

At one of the Army Service Corps Depôts—1,400 men—the only place available for our dental office was an old dirty unused kitchen in which we put two chairs, etc. As this was situated under the then unique bathroom, we constantly had to wade into the place, there being an inch of water on the floor, this having come through the ceiling. This flood was caused by the leaking bath supplying sufficient means to transform our somewhat miserable surroundings into a hydro-dental department of the Medical Service—rather a novel experience!

Being British Red Cross men, we had no military standing, and consequently no orderlies, unless we wished to pay for them; therefore during our busy hours of practical work, I had to do their work as well as my own, which, as you can imagine, was not very agreeable. However, here as elsewhere, after our united efforts had produced their effects, we were helped to the utmost limit by our Colonel and his staff.

Whether at the B. R. C. S. headquarters, at our Auxiliary Hospital, Hôtel Astoria, or at the A. S. C. dépôt St. Denis or No. 4 General Hospital, Versailles, the same final appreciation of our work was manifested, both by officers and men and to such an extend was this true, that finally we were recognized Acting Army Dental Surgeons of Paris and the surrounding districts.

The nature of our work was the ordinary routine of daily practice; fillings, extractions, prophylactic and conservative work, an enormous number of dentures being supplied. Jaw cases were sent on to Rouen, where a huge dental base was finally established and maintained by the R. A. M. C.

Our patients came from the Army Ordnance Corps; the staff and nurses from Royaumont and Villers-Cotterets; the Royal Flying Corps Paris Headquarters; their mechanics from Villacoublay, Buc, Clichy and Meudon; the cadets from the British Flying School at Vendôme; officers and orderlies from the Supreme War Council at Versailles; Canadians, Australians and other overseas men when on leave in Paris; the women helpers in the canteens and huts; men from the Forestry Corps; the Intelligence and Military missions in Paris; the Military Police and the Military Post-Office men.

In fact the B. R. C. S. Paris branch dental surgeons did from October, 1914, to June 1, 1919, all the work which otherwise would necessarily have been done by the Army Dental Surgeons.

### DRS. GEORGES AND HENRI VILLAIN

Great praise is due to two French graduates of the University of Pennsylvania established in Paris, Drs. Georges and Henri Villain. Dr. Georges Villain at the outbreak of hostilities, like all young French dentists, was sent to fulfill duties totally estranged from dentistry: ambulance bearers, orderlies in hospitals, etc., etc. After strenuous efforts on his part and those attached as directors to the Ecole Dentaire de Paris, the French dental surgeons were attached to different regiments without any official rank, and also without instruments or outfit. Their persistent endeavors to prove the value of their services, given as you can understand under great difficulties, forced the attention of the Army and Government authorities, and thanks to the intelligent and energetic Minister, who was director of the whole medical organization, they finally enjoyed equivalent positions and rank to the Army Medical men, and formed centers in Paris, Lyons, Bordeaux, Toulouse for general dental treatment, supplying of dentures. Facio-maxillary centers were established, in which the ingenious skill and original initiative, so natural to the French as a race, soon became the admiration of their colleagues in the Allied forces, and where they learned many valuable lessons in that special order.

Their beginnings were perhaps worse than ours, their triumph all the more worthy of our admiration. I would cite the names of many of their chief workers, but they, not holding American diplomas, I have to leave them, as I gather that with that general largeness of professional spirit, which is the signal feature of American Dental Associations and members, you extend to those of us foreigners who have enjoyed the advantage of completing our professional education in America a coequivalent as your own citizens.

In conclusion I would like to say that the rôle of the American dentist in Paris during the great war has been that of compelling recognition of the value of the dentist to the communities in general, and to the Army and Navy particularly. His endeavors, his skill, his patient adaptability, his personality and dexterity have made those who were inclined to ignore his use, finally place him on an equal plane with themselves and he was worthy of being there.

Prior to sitting down I wish to draw from a remarkable and wonderfully documented report which Dr. George Hayes is publishing, after presentation in Washington, a lesson of the use the dentist can be to the general surgeon when numerous cases of injuries to the face and jaws arrive for treatment; for instance, the result of the dentist's observation is the fact, which the general surgeons could not perceive, that

REPORT OF DR. GEORGE HAYES

"A bony union or partial ossification which draws an appreciable number of useful teeth out of occlusion should be reduced either by mechanical spreading, or by surgical section and insertion of bone-graft, either condition being equal.

"Lack of appreciation of proper articulation of the teeth on the part of the general surgeon led, in many instances early in the war, to bone suturing, regardless of the amount of missing substance, with resultant deformity and impossibility of mastication.

"The treatment of these cases requires not only general surgical qualifications but a thorough practical knowledge of dentistry, including orthodontics, and should be undertaken either by a dental surgeon in co-operation with a general surgeon, or preferably by an oral surgeon, meaning thereby a dental surgeon qualified in general surgery.

"The training and practice of dentistry, which involves the habitual use of delicate instruments, precision and accuracy in the fitting of pieces, and dexterity in operating in the mouth, make more of a qualification than the necessary experience in the application of general surgical principles which, in addition to dental education and training, may readily be acquired by specialization."

Also, to call to your notice the treatment of salivary fistulæ formed after extensive injuries to the face:

"The mandible was firmly bound in occlusion by four-tailed bandages, the patient instructed not to talk or smoke, and subjected to a strict regime of pure milk, followed by careful rinsing of the mouth for twenty-one days. In one case success was retarded owing to the addition of sugar or some aromatic flavoring to make the milk more palatable. It was found that this simple addition provoked a decided increase in the flow of saliva, causing a reopening of the granulating surface."

Gentlemen, I deeply appreciate your patient hearing.—*Items of Interest.*

